# Safeguarding community-centred global health research during crises

**DOI:** 10.1136/bmjgh-2023-013304

**Published:** 2023-08-02

**Authors:** Thilini Agampodi, Hasara Nuwangi, Sonali Gunasekara, Asitha Mallawaarachchi, Helen P Price, Lisa Dikomitis, Suneth Agampodi

**Affiliations:** 1 Department of Community Medicine, Faculty of Medicine and Allied Sciences, Rajarata University of Sri Lanka, Saliyapura, Sri Lanka; 2 School of Life Sciences, Keele University, Newcastle-under-Lyme, Staffordshire, UK; 3 Kent and Medway Medical School, University of Kent and Canterbury Christ Church University, Canterbury, UK; 4 New Initiatives, International Vaccine Institute, Gwanak-gu, Seoul, Korea (the Republic of)

**Keywords:** COVID-19, Health services research, Cutaneous leishmaniasis, Qualitative study, Health policy

**Summary box**

- Global health researchers face multiple challenges in proceeding with research programmes during crises, including ethical and safety questions, equitable participation of community members and the collection of robust data.
- In Sri Lanka, the multicountry global health programme ECLIPSE adopted innovative research methods in a context dictated by pandemic conditions, and strengthened by community engagement and involvement (CEI), to achieve its goals, provides a model for global health researchers working in crisis settings.
- Following the government regulations in combination with scientific guidelines, closely monitoring the pandemic and timely prediction, adopting a robust CEI approachat the early stages of research and using innovative methods that moves beyond virtual mode can help navigation of research without disruption.
- Incorporated crisis preparedness and alternative plans focusing on encouraging the use of CEI in grant proposal development by researchers and a the demand of global health research funders on these key aspects would enhance the ability of research programmes to sustain during crises.

## Introduction

Global health researchers encounter challenges in conducting research during crises, including pandemics, natural disasters and humanitarian conflicts.^1 2^ External crises often arise without prior notice and disrupt well-planned research. It is difficult to continue research activities under these circumstances, particularly when researchers and communities are at risk.^3^ Furthermore, community engagement and involvement (CEI), a crucial element in decolonised global health research,^4^ can become particularly difficult, as the community members’ primary focus may be on survival and acquiring basic needs, which must be a priority above commitment and participation in research. Conducting research in a context of crisis imposes concerns about ethical, credible and equitable research.^5 6^

The COVID-19 pandemic had a significant impact on global health research, particularly in low-income and middle-income countries (LMICs). Both funding acquisition and scholarly output in LMICs were affected.^2^ Collaborative research relied on virtual communication platforms, and alternative data collection mechanisms, such as online questionnaires and telephone interviews. However, the validity, reliability and generalisability of such datasets are still subject to extensive discussion.^7^ Populations without reliable internet access and electronic devices were often excluded from participation, which further exacerbated social inequity, particularly in disadvantaged rural communities.^8 9^

Here, we share the experience of the Sri Lankan team of the multicountry global health research programme ECLIPSE. We highlight three aspects that will inform the global scientific community in safeguarding research during crises: (1) positioning the research within the crisis context; (2) using CEI for ongoing research and (3) innovating methods and moving beyond the virtual mode.

## The ECLIPSE programme

ECLIPSE is a multicountry global health research programme with the full title: ‘Empowering people with Cutaneous Leishmaniasis: Intervention Programme to improve the patient journey and reduce Stigma via community Education’. ECLIPSE is strongly underpinned by CEI in underserved communities in Brazil, Ethiopia and Sri Lanka.^10^ The programme employs a range of methodological approaches to address knowledge gaps in CL and implement interventions to reduce disease-associated stigma and improve the patient journey. However, restrictions during the COVID-19 pandemic had a direct impact on the backbone ethnographic component of the ECLIPSE programme, in which we aimed to explore culture, health contexts and CL-related problems. Given that mobile and online platforms fail generating research data in rural areas in Sri Lanka,^11^ we adopted and implemented innovative research methods to achieve the research goals, providing a model for global health researchers.

## The ECLIPSE Sri Lanka experience during the pandemic

**Figure 1:**
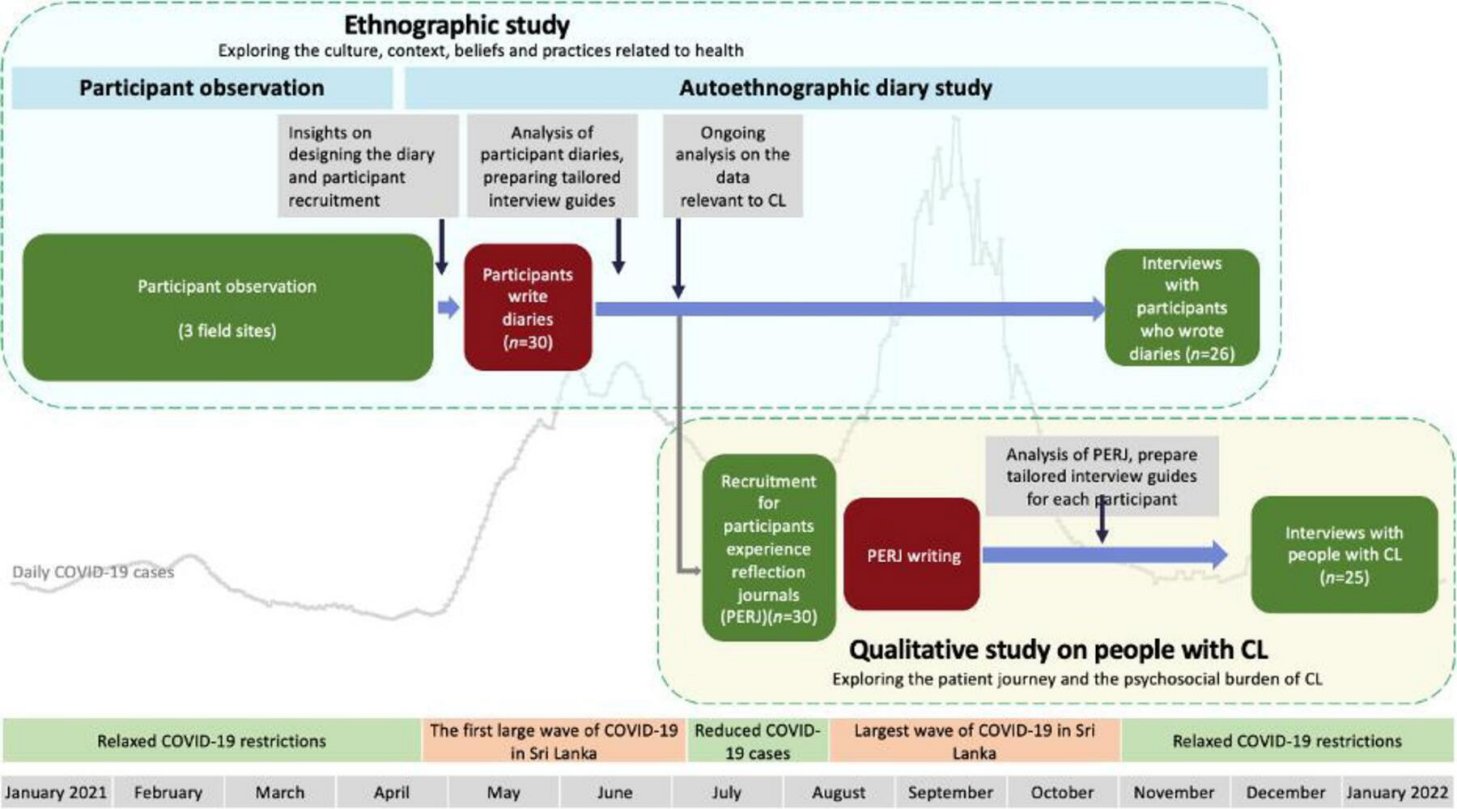
Field research on cutaneous leishmaniasis (CL) during the two large outbreaks of COVID-19 in Sri Lanka.

The ECLIPSE Sri Lanka 6-month ethnographic study was planned from September 2020 onwards. However, as part of the government response to the pandemic, there was a complete country lockdown, police curfews and movement restrictions, and it was impossible to commence CEI-oriented research at that time. Hence, the team needed to wait (despite the timeline of the main project) until December 2020, when a degree of normalisation returned, to establish community stakeholder groups that engaged in designing the study with the researchers. With due partnership built on trust, we initiated ethnographic fieldwork in January 2021. The researchers conducting participant observation were living in ECLIPSE communities. However, the prediction by leading Sri Lankan epidemiologists of the emergence of a large COVID-19 outbreak in April 2021 necessitated modifications to our ethnographic approach. Recognising the scientific validity of these predictions, we planned alternative ethnographic methods as a risk-reducing measure to sustain the ECLIPSE programme (see figure 1).

Within a span of 2 weeks, the research team responded to changing the methodological approach from conventional participant observation to a community-led autoethnographic diary study. Community members were actively involved and were trained as community researchers to ensure the continuity of the ethnographic study during the period when ECLIPSE researchers could no longer live in the communities. We established a remote monitoring system underpinned by CEI principles, wherein community members were included as team members. These trained community researchers had, by the nature of living in the field site, continuous access to the study participants.

When Sri Lanka experienced its first large COVID-19 outbreak, between April and June 2021, the research team had already implemented an alternative methodology to ensure the uninterrupted progress of their research efforts. Notably, the alpha variant of SARS-CoV-2 was responsible for the surge in COVID-19 cases during the study period. Foreseeing the window of opportunity between the two waves (alpha and beta variants) the team formulated its subsequent actions by adopting a strategy of collecting diaries with minimal in-person contact and engaged in ongoing iterative analysis to inform the upcoming stage. The next phase of the study involved conducting in-person semistructured interviews with individuals with CL. The research team developed a Participant Experience Reflection Journal (PERJ), in which participants could document their experiences and reflections on the CL patient journey.^12^ Following the relaxation of certain restrictions at the end of July 2021, we recruited participants for the PERJ (figure 1). Visits by researchers to field sites posed potential risks to all parties and were not well received by communities in some instances. Therefore, in order to minimise the number of visits to communities but also maintain progression, we executed a CEI approach that involved community members in designing, participant selection and distribution of PERJ.

The second and most significant wave of the COVID-19 pandemic in Sri Lanka occurred between August and October 2021. The research team worked on comprehensive analysis of participant diaries and developing interview guides for postdiary interviews. Concurrently, individuals with CL completed the PERJ mediated by the remote monitoring system. In October 2021, the Sri Lankan government lifted restrictions following the reduction in the number of cases and the communities were willing to have researchers back in the field. We conducted ‘postdiary’ and postjournal’ interviews^13^ with relevant participants from December 2021 to January 2022.^12^

## Positioning the research activities within the crisis context

To conduct effective global health research, especially during times of crisis, research teams must have a comprehensive understanding of the political, economic and societal contexts and community perceptions, behaviours, policies, and regulations that may affect their work. Appropriate positioning of research activities within this context requires a scientific approach that responds to challenges while maintaining the rigour of the research methods. Our experience with ECLIPSE demonstrates the importance of understanding and evaluating the complex variables involved in field research to ensure that research activities are appropriately positioned within a given context.

## Using CEI for ongoing research during a crisis

CEI refers to various approaches in which people are included in health research decision-making, de-centred participatory planning, knowledge coproduction and resource sharing.^14^ Apart from the clear barriers to CEI during pandemics, studies on the positive impacts of CEI on research during crises^15^ are scarce. We demonstrated that a well-established CEI approach is still feasible and remains highly valuable for global health research programmes during times of crisis. The ECLIPSE Sri Lanka country study benefited from the CEI approach that had been embedded into the study prepandemic. As a programme implementing CEI from the start, community owners were involved in the decision-making, selecting feasible methods, the timing of contact, facilitating community work, engaging in data collection and monitoring, and linking the research team’s appropriate crisis-related activities for the community’s benefit. Hence, trust building, two-way communication and partnership are integral components of research during crises.

## Innovation and moving beyond the virtual mode

In light of these challenges of virtual data collection methods,^8 9^ it is crucial for researchers to embrace innovative approaches to data collection and analysis as well as to restructure, replan and use alternative methods. While there may not be an all-in-one solution for all research approaches, it is essential for researchers to have contingency plans and an open and dynamic mindset to adapt to the research process during a crisis. It is imperative that researchers adopt a pragmatic approach that leverages both traditional and innovative methods. Our experience demonstrates the need for researchers to be flexible, adaptable and innovative in embracing a range of alternative methods, while remaining mindful of the limitations and potential biases inherent in each approach.

## Way forward

Global health research programmes are integral in addressing the healthcare needs of disadvantaged societies, knowledge generation and capacity building. However, the limited evidence surrounding adapting methodological approach in order to meet the same objectives and discussion of such alternative research methods and crisis readiness highlights the need for increased attention of funding bodies in study proposal and protocol development.

To address this gap, funding organisations must prioritise the demand for emergency preparedness in research programmes particularly given ongoing environmental, humanitarian and biomedical challenges. Risk management and alternative crisis preparedness plans need to be incorporated into research proposals, encouraging the use of CEI in the early stages of research. Additionally, a platform for sharing novel methods governing equity and inclusion should be developed and shared to enhance the knowledge, creativity and skills of researchers. It is time to recognise the importance of crisis preparedness in research programmes, ensuring the equitable and inclusive engagement of communities in the research process.

## Data Availability

Data pertaining to this manuscript is included in the manuscript.
